# Henry Gas Solubility Optimization Algorithm based Feature Extraction in Dermoscopic Images Analysis of Skin Cancer

**DOI:** 10.3390/cancers15072146

**Published:** 2023-04-04

**Authors:** Marwa Obayya, Adeeb Alhebri, Mashael Maashi, Ahmed S. Salama, Anwer Mustafa Hilal, Mohamed Ibrahim Alsaid, Azza Elneil Osman, Amani A. Alneil

**Affiliations:** 1 Department of Biomedical Engineering, College of Engineering, Princess Nourah bint Abdulrahman University, Riyadh 11671, Saudi Arabia; 2 Department of Accounting, Applied College, King Khalid University, Mohail Asser 63311, Saudi Arabia; 3 Department of Software Engineering, College of Computer and Information Sciences, King Saud University, Riyadh 11543, Saudi Arabia; 4 Department of Electrical Engineering, Faculty of Engineering & Technology, Future University in Egypt, New Cairo 11845, Egypt; 5 Department of Computer and Self Development, Preparatory Year Deanship, Prince Sattam bin Abdulaziz University, Al Kharj 11942, Saudi Arabia

**Keywords:** skin cancer, computer aided diagnosis, dermoscopic images, MAFNet, metaheuristics

## Abstract

**Simple Summary:**

Early diagnosis of skin cancer is vital for providing effective treatment for patients. Dermoscopy is a non-invasive approach that utilizes specific equipment to examine the skin and is helpful in determining the specific patterns and features that might confirm the existence of skin cancer. Recently, Machine Learning (ML) algorithms have been developed to analyze dermoscopic images and classify such images as either benign or malignant. Convolutional Neural Networks (CNNs) and other ML techniques, such as the Support Vector Machine (SVM) and Random Forest classifiers, have been used in the extraction of the features from the dermoscopic images. The extracted features are then used to classify the dermoscopic images as either benign or malignant. Therefore, the current study develops a new Deep Learning-based skin cancer classification method for dermoscopic images, which has the potential to improve the accuracy and efficiency of the skin cancer diagnosis process and produce better outcomes for the patients.

**Abstract:**

Artificial Intelligence (AI) techniques have changed the general perceptions about medical diagnostics, especially after the introduction and development of Convolutional Neural Networks (CNN) and advanced Deep Learning (DL) and Machine Learning (ML) approaches. In general, dermatologists visually inspect the images and assess the morphological variables such as borders, colors, and shapes to diagnose the disease. In this background, AI techniques make use of algorithms and computer systems to mimic the cognitive functions of the human brain and assist clinicians and researchers. In recent years, AI has been applied extensively in the domain of dermatology, especially for the detection and classification of skin cancer and other general skin diseases. In this research article, the authors propose an Optimal Multi-Attention Fusion Convolutional Neural Network-based Skin Cancer Diagnosis (MAFCNN-SCD) technique for the detection of skin cancer in dermoscopic images. The primary aim of the proposed MAFCNN-SCD technique is to classify skin cancer on dermoscopic images. In the presented MAFCNN-SCD technique, the data pre-processing is performed at the initial stage. Next, the MAFNet method is applied as a feature extractor with Henry Gas Solubility Optimization (HGSO) algorithm as a hyperparameter optimizer. Finally, the Deep Belief Network (DBN) method is exploited for the detection and classification of skin cancer. A sequence of simulations was conducted to establish the superior performance of the proposed MAFCNN-SCD approach. The comprehensive comparative analysis outcomes confirmed the supreme performance of the proposed MAFCNN-SCD technique over other methodologies.

## 1. Introduction

Melanoma is the most severe type of skin cancer that appears on any part of the skin or near a mole. This skin cancer is characterized by an uncontrollable growth of the cells without any apoptosis [1]. In this scenario, such cells of the body parts turn out to be tumorous and start spreading to other parts of the party. Unlike the rest of the skin cancer types, such as basal cell carcinoma and squamous cell carcinoma, melanoma is less common in nature. However, melanoma is highly dangerous compared to the rest of the skin cancer types, as it spreads to distinct body parts if left untreated or undiagnosed in its early stages. Melanoma spreads rapidly across the body and affects almost all body parts [2]. The dermatologists utilize photographic or microscopic instruments to see the additional details that are relevant to lesions. When diagnosing skin cancer, the clinician refers the individual to a tumor expert who performs surgery on the lesions [3]. Dermoscopy is a microscopic technique that inspects the surface of the skin. This technique is utilized to distinguish benign lesions from malignant ones based on the captured images without removing the skin and other maddening tests. This analytical procedure is conducted completely based on the oncologist’s expertise and experience [4]. Such a scenario pushed the current study authors to develop a computer-aided technique by employing the dermoscopic images and displaying the outcomes as assisting apparatuses for dermatologists. Various studies have been conducted so far to attain superior outcomes in the disease diagnosis process.

Numerous methods have been modeled so far for the automatic detection of melanoma-affected skin parts [5]. At first, handcrafted features-related methods were presented for diagnosing melanoma. But, such methods did not yield good outcomes due to dissimilarities in the color, shape, and size of the melanoma moles [6]. Then, segmentation-related methods such as the Iterative Selection Thresholding (ISO) and adaptive thresholding were proposed to enhance the detection accuracy of such automated mechanisms. Such methods tend to work on the segmented part of the melanoma called ‘RoI’ [7]. DL-related techniques have gained more popularity in medical imaging and diagnostics processes in recent years. In techniques such as the CNN, a small portion of the images, with melanoma-affected portions, is considered to train the automatic identification mechanism [8]. There exist numerous potential applications for deep unsupervised learning-based feature extraction from these images, such as object detection, image classification, image retrieval, anomaly detection, generative modeling, etc. Such methods execute the segmentation process on the test images that are related to the trained method. The DL-related techniques exhibit superior performance in detecting and segmenting melanoma images than the handcrafted feature-related methods [9]. Such procedures can automatically calculate the complicated and representative feature set. Furthermore, the DL methods can also easily trace the skin moles of different sizes in the occurrence of noise, blur, the incidence of light, color variations, and intensity [10].

The current research article develops an Optimal Multi-Attention Fusion Convolutional Neural Network-based Skin Cancer Diagnosis (MAFCNN-SCD) technique for the diagnosis of melanoma cancer from dermoscopic images. In the presented MAFCNN-SCD technique, the data pre-processing is performed initially. Next, the MAFNet method is enforced as a feature extractor with Henry Gas Solubility Optimization (HGSO) algorithm as a hyperparameter optimizer. Finally, the Deep Belief Network (DBN) method is exploited for the detection and classification of skin cancer. A sequence of experiments was conducted to validate the improved performance of the proposed MAFCNN-SCD approach. The key contributions of the current research work are listed herewith.

- An automated MAFCNN-SCD technique has been proposed in this study with pre-processing, MAFNet-based feature extraction, DBN classification, and HGSO-based hyperparameter tuning processes for skin cancer detection and classification. To the best of the authors’ knowledge, the proposed MAFCNN-SCD model is the first of its kind in this domain.
- The authors employed MAFNet as a feature extractor with DBN as a skin cancer detection and classification classifier.
- The hyperparameter optimization of the MAFNet model, using the HGSO algorithm with cross-validation, helped to boost the predictive outcomes of the proposed MAFCNN-SCD model for unseen data.

## 2. Literature Review

Shorfuzzaman [11] presented an interpretable CNN-based stacked ensemble structure for the detection of melanoma skin tumors at earlier stages. In this study, the Transfer Learning (TL) model was employed in the stacked ensemble framework, whereas a distinct number of CNN sub-models that apply similar classifier tasks were also gathered. A novel approach called meta-learner was employed in the prediction of every sub-model, and the last prediction outcomes were attained. Bhimavarapu and Battineni [12] intended to integrate the DL techniques for the automatic classification of melanoma in dermoscopic images. A Fuzzy-based GrabCut-stacked CNN (GC-SCNN) technique was validated using the trained images. The image extraction feature and the lesion classifier were leveraged, and the model’s efficacy was tested using distinct openly-accessible databases. The purpose of the study, conducted by Lafraxo et al. [13], was to automate the procedure of classifying the dermoscopic images comprising skin lesions as either benign or malignant. Thus, an enhanced DL-based solution with CNN was presented in this study. Data augmentation, regularization, and dropout were performed to avoid the over-fitting issue that is generally experienced in the CNN technique.

Banerjee et al. [14] examined a DL-based YOLO technique on the basis of the application of DCNNs. This technique was used to detect melanoma in digital and dermoscopic images. The authors suggested a faster and a precise outcome related to the typical CNNs. But, particular resourceful models were infused under two stages of segmentation. This segmentation is created by combining a graph model using a minimal spanning tree model and an L-type fuzzy number. The latter is related to approximation and mathematical extraction of the actual affected lesion regions in the feature extraction method. Daghrir et al. [15] established a hybrid system for melanoma skin tumor classification, and the method was employed for examining a few suspicious lesions. The presented method was dependent on the prediction of three distinct approaches, such as CNN and two typical ML techniques. These techniques were trained with a group of characteristics that explain the texture, border, and color of the skin tumor.

Tan et al. [16] presented an intelligent Decision Support System (DSS) for skin tumor classification. Specifically, the authors integrated the medically-essential features such as asymmetry, color, border irregularity, and other such dermoscopic structural features with the texture extraction features using the Histogram of Oriented Gradients (HOG), Grey Level Run Length Matrix and LBPs functions for tumor representations. Afterward, the authors presented two improved PSO techniques for the optimization of the features. In literature [17], a Hybrid DL (HDL) system was proposed fusing the sub-band of 3D wavelets. It was a non-invasive and objective system that was used for the inspection of skin images. During the primary phase of the HDL system, an easy Median Filter (MF) was utilized to remove unwanted data like noise and hair. During the secondary phase, the sub-band fusion method was used, and the 3D wavelet transform was executed to obtain the textural data in the dermoscopic images. In the last phase, the HDL system carried out a multiclass classification with the help of the fused sub-band.

Though several models have been proposed in the literature, the existing models do not focus on the hyperparameter selection process. This is a crucial process as it mostly influences classification performance. The hyperparameters such as epoch count, batch size, and learning rate selection are essential to attain effectual outcomes. Since the trial-and-error method for hyperparameter tuning is a tedious and erroneous process, the metaheuristic algorithms are applied. Therefore, in this research work, the HGSO algorithm is employed to select the parameters for the MAFNet model.

## 3. The Proposed Model

**Figure 1:**
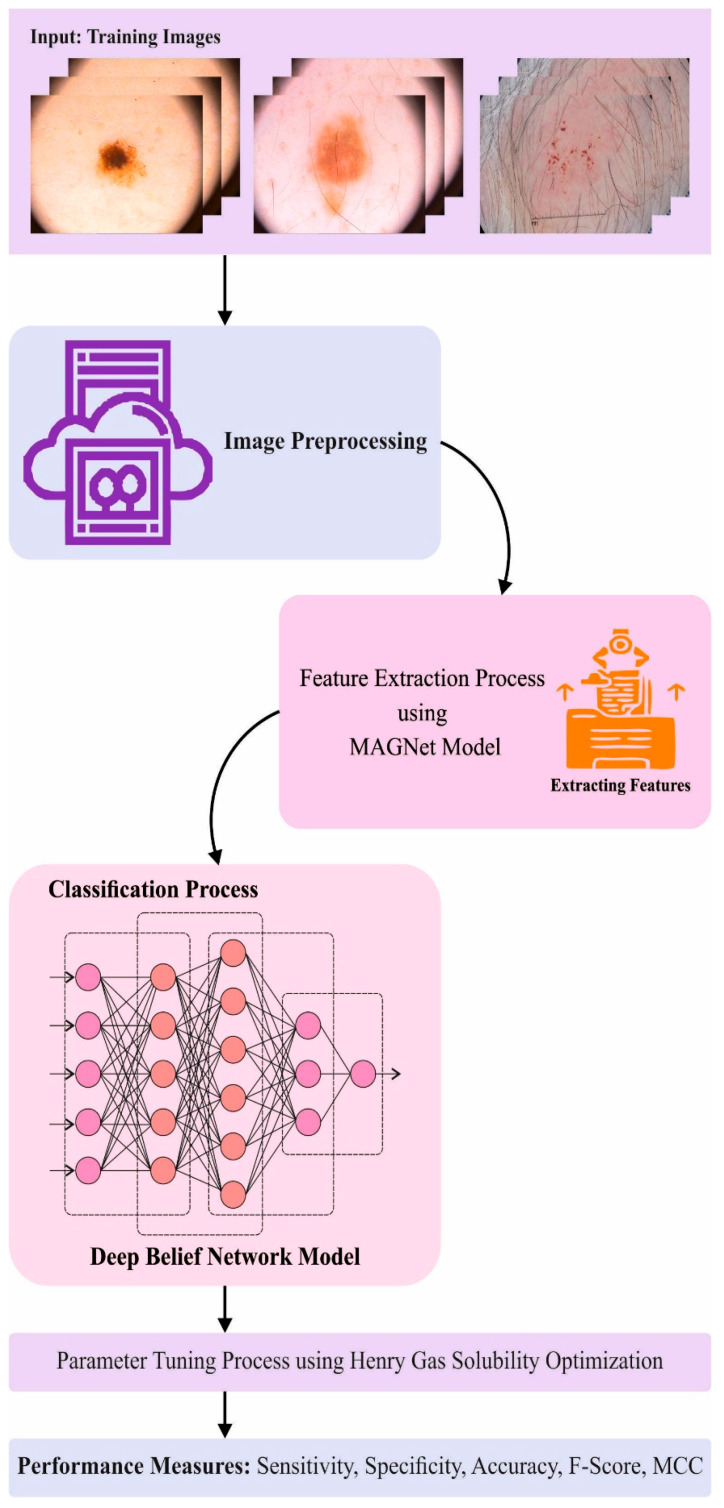
The overall process of the proposed MAFCNN-SCD system.

In this study, a new MAFCNN-SCD method has been proposed for the detection and classification of skin cancer from dermoscopic images. The major aim of the proposed MAFCNN-SCD technique is to diagnose and classify the type of skin cancers from dermoscopic images. It encompasses various stages such as image pre-processing, MAFNet feature extraction, HGSO hyperparameter tuning, and DBN classification. Figure 1 defines the overall procedure of the proposed MAFCNN-SCD system.

### 3.1. Image Pre-Processing

In the presented MAFCNN-SCD approach, the data is pre-processed initially by following the Weiner Filter technique. The corrupted image is determined as $\hat{I}\left(x,y\right)$, whereas the local mean is denoted by $\hat{\mu_{L}}$ on a pixel window, the noise variance with the entire value is demonstrated by $\sigma_{y}^{2}$, whereas the local variance in the window is designated by ${\hat{\sigma }}_{y}^{2}$. Then, the probable method of denoising the image is shown below [18]:

(1) $$\hat{\hat{I}}=\hat{I}\left(x,y\right)-\frac{\sigma_{y}^{2}}{{\hat{\sigma }}_{y}^{2}}\left(\hat{I}\left(x,y\right)-\hat{\mu_{L}}\right)$$

Here, when the noise variance across the image is equivalent to 0, then $\sigma_{y}^{2}=0=>\hat{\hat{I}}=\hat{I}\left(x,y\right)$. If the global noise variance is smaller, then the local variance is larger than the global variance, according to which the ratio becomes almost equal to 1.

If ${\hat{\hat{I}}}_{y}^{2}\gg \sigma_{y}^{2}$, then $\hat{\hat{I}}=Î\left(x,y\right)$. Whereas a higher local variance portrays the occurrence of the edges in the image windows [19]. In this case, when the local and global variances correspond together, the formulation progresses as follows: $\hat{\hat{I}}=\hat{\mu_{L}}as{\hat{\sigma }}_{y}^{2}\approx \sigma_{y}^{2}$.

### 3.2. Feature Extraction Model

**Figure 2:**
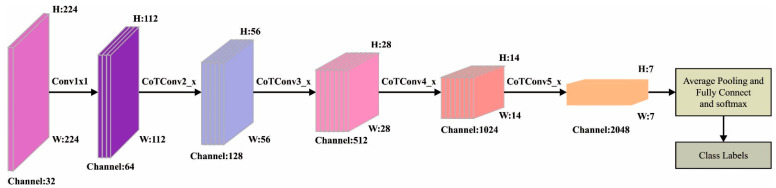
Architecture of MAFNet.

In this study, the MAFNet method is applied as a feature extractor. MAFNet encompasses 1×1×1 convolution layers [20]; four convolution models exist in the middle, whereas three convolution structures exist across all the modules. The convolution module is disseminated in a symmetrical structure like [2,2,2,2]. At last, the FC layer is present with a total of 26 convolution modules [21,22]. Afterward, the images are fed as input, and the 1×1 convolutional process is initially performed. Next, four convolution models are passed over four convolution models. The 3×3 convolutional operation is substituted by the Contextual Transformer (CoT) blocks in the original ResNet convolution blocks. After the preceding convolution, pooling, and excitation operations, the features extracted are then fed as input to the FC layers that perform the role of “classification” in the CNN technique [23]. The architecture of the MAFNet model is shown in Figure 2. The FC layers perform the “classification” process in the CNN method that incorporates the preceding and extremely-abstracted feature and maps the learned feature to a sample space. It also employs the Softmax function to evaluate the probability of classification. At last, the output of the classifier outcome is given as follows.

(2) $$Softmax\left(z_{j}\right)=\frac{e^{z_{i}}}{\sum_{i=1}^{n}e^{z_{i}}},$$

Equation (2) demonstrates the number of classes, $z_{j}$ represents the output value of the j-th node, $z_{i}$ indicates the output value of the i-th node, and e shows the natural constant, which can be determined as follows.

(3) $$L_{CE}=-\overset{N}{\underset{i=1}{\sum }}l_{i}logp_{i,}$$

In Equation (3), $l_{i}$ refers to the unique thermal encoding of the tag $i(i\in \left(0,\cdots ,N-1\right)$ and N indicates the number of tags; when the target label is i, then $l_{i}=1$, whereas the rest of the labels are equivalent to 0, and $p_{i}$ denotes the predictive probability of the i-th label, i.e., the Softmax value.

It must be noted that the lowest learning rate might result in slower convergence, and the largest learning rate might result in a constant oscillation of the loss functions. In order to construct a model with high accuracy and optimum parameters followed by its quick training, a dynamic-learning-rate approach is utilized. This helps in adjusting the learning rate per 30 epochs, for which the formula is given below [24].

(4) $$lr=lr_{0}\times 0.1^{\frac{epoch}{30}},$$

In Equation (4), lr denotes the present learning rate, $lr_{0}$ indicates the primary learning rate, and epoch represents the overall number of training rounds.

In this study, the HGSO algorithm is applied as a hyperparameter optimizer. Hashim et al. [25] presented the HGSO technique, which is a newly-found physics-inspired optimization algorithm. This technique has been constructed based on Henry’s gas law which defines the rules for the solubility of a gas in a liquid. In general, temperature and pressure are two major elements that significantly impact the outcome of solubility. In regard to pressure, the ability of a gas to become solvable in the liquid increases when the pressure increases. At high temperatures, the solubility of the solid increases. On the other hand, gases cannot dissolve. By utilizing these two significant characteristics, the HGSO approach comprises eight steps, as listed herewith. Initially, the values of Henry’s constant per group j $\left(H_{j}\left(t\right)\right)$, the number of gases (population), position, and the partial pressure $P_{i,j}$ of the gas i at every group j are generated and are given as a mathematical form herewith.

(5) $$X_{i}\left(T+1\right)=X_{min}+r\times \left(X_{max}-X_{min}\right),$$

In Equation (5), $X_{i}$ signifies the location of the i-th gas in population N, r is determined by the chaotic number between 0 and 1, and $X_{min}$ and $X_{max}$ denote the bounds of the search space and (*t*) shows the iteration.

(6) $$H_{j}\left(t\right)=l_{1}\times rand{\left(0_{t}1\right)}_{t}P_{i,j}=l_{2}\times rand{\left(0,1\right)}_{t}C_{j}=l_{3}\times rand\left(0,1\right),$$

In Equation (6), $l_{1}$ to $l_{3}$ indicate the constant numbers that correspond to the values between 5 × 10^−2^, 100 and 1 × 10^−2^.

Next, the clustering process is executed in which the population of the gas is categorized according to the type of gas. In all the groups, each gas has a similar $H_{j}.$

Then, the evaluation process is conducted to determine the most suitable gas from every cluster j that attains the maximum equilibrium location over the rest of the gases. In this stage, the ranking is used to find the most suitable gas among the entire swarm [26].

Henry’s coefficient is expressed in the following equation.

(7) $$H_{j}\left(t+1\right)=H_{j}\left(t\right)\times e^{\left(-C_{j}\left(\frac{\frac{1}{T\left(t\right)}1}{T^{\theta }}\right)\right)},T\left(t\right)=e^{\left(-\frac{\tau }{iter}\right)}$$

In Equation (7), $H_{j}$ demonstrates the coefficient of Henry’s gas rules in all the groups j, T characterizes the temperature, and $T^{\theta }$ describes the fixed quantity with a value of 298. Furthermore, iter signifies the overall iteration count. The solubility updating formula is given below in which $S_{i,j}$ demonstrates the solvability of a gas i in all the groups j, $P_{i,j}$ characterizes the parochial pressure on gas i in all the groups j, and K denotes a fixed value.

(8) $$S_{i,j}\left(t\right)=K\times H_{j}\left(t+1\right)\times P_{i,j}\left(t\right).$$

Step 6 characterizes a formula to upgrade the location of all the gases in Equations (9) and (10):

(9) $$X_{i,j}\left(t+1\right)=X_{i,j}\left(t\right)+F\times r\times \gamma \times \left(X_{i,bes\tau }\left(t\right)-X_{i,j}\left(t\right)\right)\;+\;F\times r\times \alpha \times \left(S_{i,j}\left(t\right)\times X_{i,best}\left(t\right)-X_{i,j}\left(t\right)\right)$$

(10) $$\gamma =\beta \times exp(-\frac{F_{best}\left(t\right)+\varepsilon }{F_{i,j}\left(t\right)+\varepsilon })t\varepsilon =0.05$$

In Equation (10), $X_{i,j}$ characterizes the condition of all the gases i in all the groups j, r denotes the random numbers between 0 and 1, whereas τ denotes the iteration time, and $X_{i,best}$ illustrates the most suitable gas i in all the groups j. Further, $X_{best}$ characterizes the most suitable gas amongst the entire population [27]. In addition to these, γ signifies the ability of all the gases j in all the groups i to interact through the gases in its group, and α illustrates the effect of the remaining gases on i in every group j and takes the value of 1. Additionally, a certain number is allocated to β. $F_{i,j}$ shows the fitness of all the gases i in all the groups j at the same time, and $F_{best}$ demonstrates the fitness of the most suitable gas in the entire population. The formula to prevent the local optima situation is given below.

(11) $$N_{w}=N*(rand\left(\left(C_{2}-C_{1}\right)+C_{1}\right),C_{1};C_{1}=0.1,C_{2}=0.2,$$

In Equation (11), $N_{w}$ and N indicate the worst agent and the number of search agents.

Lastly, the formula for updating the location of the worst agent is given below.

(12) $$G_{i,j}=G_{Min\left(i,j\right)}+r\times {\left(G_{Max\left(i,j\right)}-G_{Min\left(i,j\right)}\right)}_{t}$$

In Equation (12), $G_{i,j}$ demonstrates the condition for all the gases i in group j, r denotes a number that is disseminated between 0 and 1, and $G_{Min\left(i,j\right)}$ and $G_{Max\left(i,j\right)}$ indicate the bounds for the algorithm. Algorithm 1 illustrates the steps followed in the HGSO approach. The HGSO method derives a Fitness Function (FF) for the enhancement of the classification outcome. It sets a positive value to designate the superior outcomes of the candidate solutions.
**Algorithm 1:** Pseudocode of HGSO Algorithm.Initialization: $i\left(1=1,2,\ldots N\right)$, number of gas kinds i,Hj,Pi,j,Cj,l1,l2, and l3. Split the population agent into a number of gas kinds (cluster) with a similar Henry’s constant value $\left(Hj\right)$. Estimate every cluster j. Obtain the more suitable gas Xi, better in all the clusters, and the better search agent Xbest. While t< maximal iteration count, do For every search agent, do Upgrade the position of each search agent through Equations (9) and (10). End for Upgrade Henry’s s coefficient of all the gas kinds based on Equation (7). Upgrade solubility of all the gases based on Equation (8). Select and Rank the amount of worst agents based on Equation (11). Upgrade the position of the worst agent based on Equation (12). Upgrade the more suitable Xi, better, and the better search agent Xbest. End while
 t=t+1 Return Xbest

In this work, the reduced classifier error rate is signified as FF as mentioned in Equation (13).

(13) $$Fitness=\frac{number\;of\;misclassified\;samples}{Total\;number\;of\;samples}*100$$

### 3.3. Skin Cancer Detection Model

In this last stage, the DBN model is exploited for the detection and classification of skin cancer. DBN is a NN that comprises numerous Restricted Boltzmann Machines (RBM) [28]. The input unit specifies the character of the original dataset, whereas the output unit specifies the label of this dataset. From the input to the output layers, the key features of the data are mined from the deep architecture via the layer abstraction process. The DBN can be accomplished by stacking numerous RBMs. The initial-layer RBM is the input of the DBN of pipeline leak detection, whereas the output is characterized by the latter, i.e., RBM Hidden Layer (HL). The DBN is processed as an MLP, and applied to the classifier, after which the LR is added to the output. The DBN model comprises certain RBMs. Every RBM has a visible layer (VL) and an HL. Consider $v={\left\{0,1\right\}}^{n}$ and $h={\left\{0,1\right\}}^{m}$ as the states of VL and HL, correspondingly. The quantity of the RBM joint configuration energy comprises biases and weights.

(14) $$E\left(v,h;\theta \right)=-\overset{n}{\underset{i=1}{\sum }}a_{j}v_{j}-\overset{m}{\underset{j=1}{\sum }}b_{j}h_{j}-\overset{n}{\underset{i=1j}{\sum }}\overset{m}{\underset{=1}{\sum }}w_{ij}v_{j}h_{j}$$

Here, $\theta =\left\{a_{j},b_{j},w_{ij}\right\}$ represents the model parameter, $w_{i_{\dot{j}}}$ indicates the weight between the hidden unit j and the visible unit $i;\;a_{j}$ and $b_{j}$ show the biases of the VL and HL, correspondingly; n and m denote the count of visible and hidden units, correspondingly. The joint likelihood equation for VL and HL is given below.

(15) $$p\left(v,h;\theta \right)=\frac{1}{Z\left(\theta \right)}exp\left(-E\left(v,h;\theta \right)\right)$$

In Equation (15), $Z\left(\theta \right)$ refers to the normalizing factor that is formulated as follows.

(16) $$Z\left(\theta \right)=\underset{v}{\sum }\underset{h}{\sum }exp\left(-E\left(v,h;\theta \right)\right)$$

Since the visible–visible and hidden–hidden cases are independent of each other, the conditional probability of this unit can be formulated using the following equation [29]:

(17) $$p\left(h_{j}=1|\theta \right)=\frac{1}{1+\exp\left(-b_{j}-\sum_{i}w_{ij}v_{i}\right)}$$

(18) $$p\left(v_{j}=1|\theta \right)=\frac{1}{1+\exp\left(-a_{i}-\sum_{j}w_{ij}h_{j}\right)}$$

**Figure 3:**
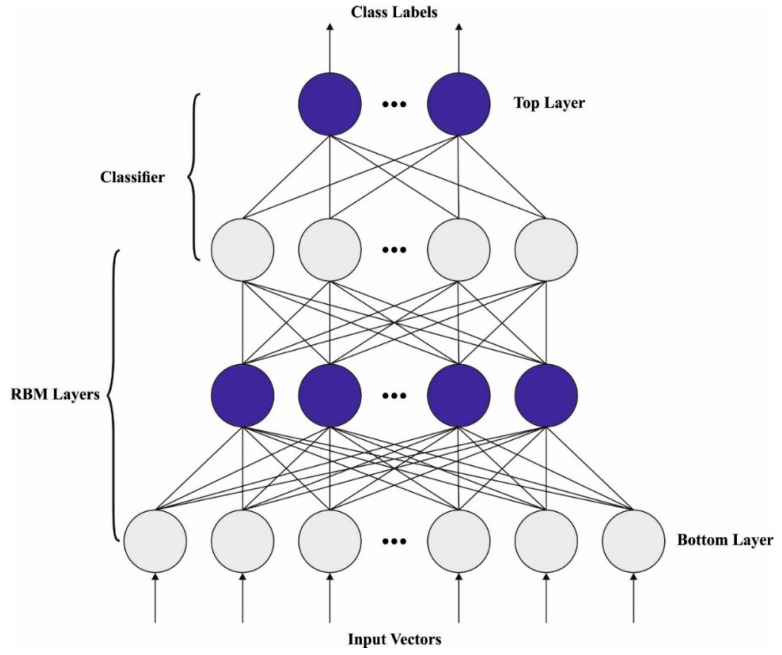
DBN structure.

The layer-wise learning mechanism of the DBN comprises three HLs. The training dataset originates from a similar pipeline with similar experimental conditions and leakage sizes. Firstly, the training dataset is transferred to the VLs on the initial RBM unit. Then, the hidden unit feeds the input dataset in the VL. At last, the VLs of the 2nd RBM unit obtain a hidden unit in the RBM [30]. The subsequent individual RBM units accomplish the exercises of the DBN structure. Figure 3 demonstrates the infrastructure of the DBN.

## 4. Performance Evaluation

The proposed model was simulated using Python 3.6.5 tool on a PC with configurations such as i5–8600 k, GeForce 1050 Ti 4 GB, 16 GB RAM, 250 GB SSD, and 1 TB HDD. The parameter settings are given herewith: learning rate: 0.01, dropout: 0.5, batch size: 5, epoch count: 50, and activation: ReLU.

### 4.1. Dataset Used

**Figure 4:**
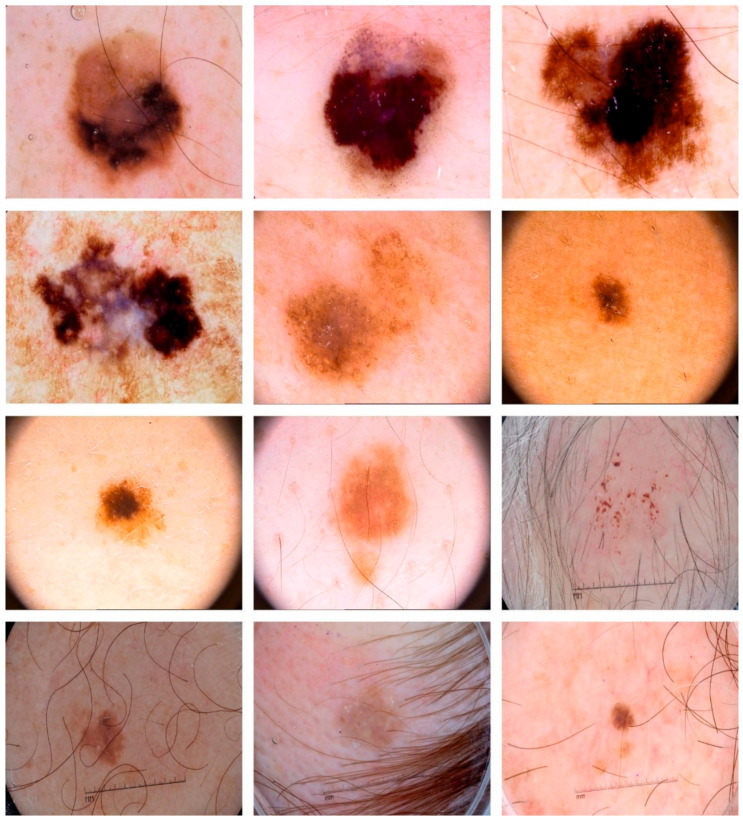
Sample images.

The presented MAFCNN-SCD model for the skin cancer classification process was validated using two benchmark databases such as the ISIC 2017 database [31] and the HAM10000 database [32]. The ISIC 2017 dataset comprises 2000 images under three classes, whereas the HAM10000 dataset holds a total of 10,082 samples under seven classes. Table 1 and Table 2 show a detailed description of the datasets under study. Figure 4 demonstrates some of the sample images from the datasets.

### 4.2. Results Analysis

**Figure 5:**
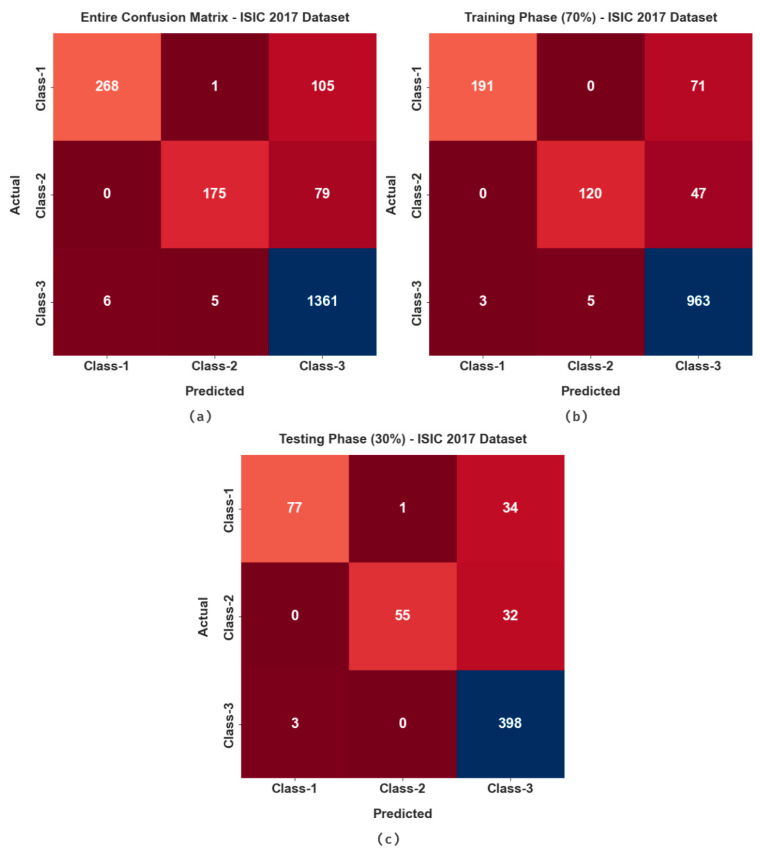
Confusion matrices of the proposed MAFCNN-SCD system under ISIC 2017 dataset. (**a**) Entire database, (**b**) 70% of TR database, and (**c**) 30% of TS database.

The confusion matrices generated by the proposed MAFCNN-SCD approach on ISIC 2017 dataset are demonstrated in Figure 5. The figure states that the proposed MAFCNN-SCD system proficiently recognized all three types of skin cancers from the applied dermoscopic images.

Table 3 portrays the overall skin cancer classification outcomes achieved by the proposed MAFCNN-SCD method on ISIC 2017 dataset. On the entire dataset, the MAFCNN-SCD method attained the average $accu_{y}$, $sens_{y}$, $spec_{y}$, $F_{score}$, and Mathew Correlation Coefficient (MCC) values, such as 93.47%, 79.92%, 90%, 85.50%, and 79.26%, respectively. Concurrently, on 70% TR database, the proposed MAFCNN-SCD approach achieved the average $accu_{y}$, $sens_{y}$, $spec_{y}$, $F_{score}$, and MCC values, such as 94.00%, 81.31%, 90.61%, 86.61%, and 80.66%, correspondingly. In parallel, on 30% of the TS database, the presented MAFCNN-SCD method achieved the average $accu_{y}$, $sens_{y}$, $spec_{y}$, $F_{score}$, and MCC values, such as 92.22%, 77.07%, 88.67%, 83.05%, and 76.23%, correspondingly.

**Figure 6:**
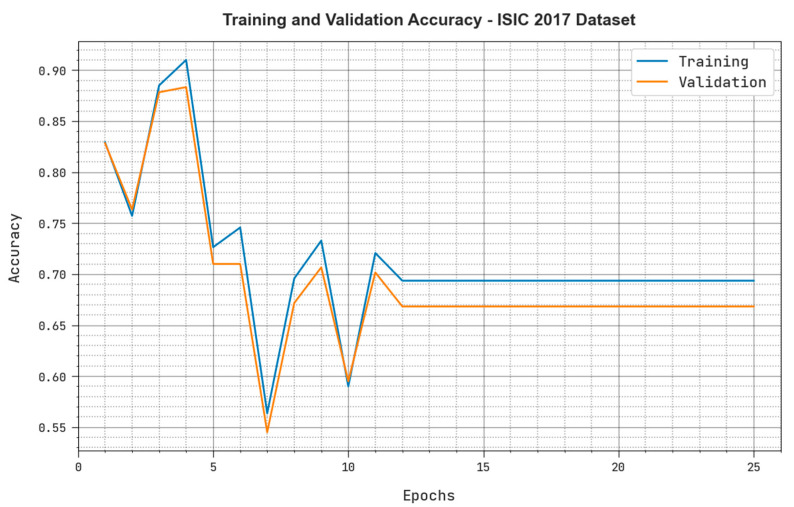
$TR_{acc}$ and $VL_{acc}$ results of the proposed MAFCNN-SCD system upon the ISIC 2017 dataset.

Both the Training Accuracy ($TR_{acc}$) and the Validation Accuracy ($VL_{acc}$) values acquired by the MAFCNN-SCD approach under ISIC 2017 dataset are presented in Figure 6. The simulation results emphasize that the proposed MAFCNN-SCD algorithm gained increased $TR_{acc}$ and $VL_{acc}$ values, while the $VL_{acc}$ values were better than the $TR_{acc}$ values.

**Figure 7:**
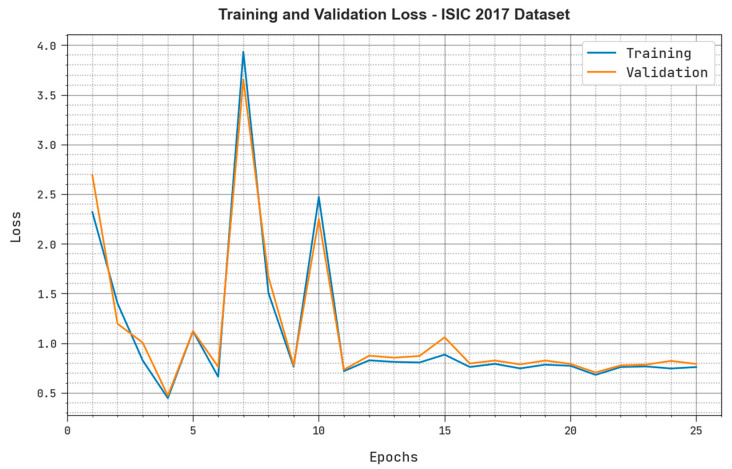
$TR_{loss}$ and $VL_{loss}$ analyses outcomes of the proposed MAFCNN-SCD system upon the ISIC 2017 dataset.

Both Training Loss ($TR_{loss}$) and the Validation Loss ($VL_{loss}$) values realized by the proposed MAFCNN-SCD system under ISIC 2017 dataset are exhibited in Figure 7. The simulation results represent that the proposed MAFCNN-SCD approach obtained the least $TR_{loss}$ and $VL_{loss}$ values, while the $VL_{loss}$ values were lesser than the $TR_{loss}$ values.

**Figure 8:**
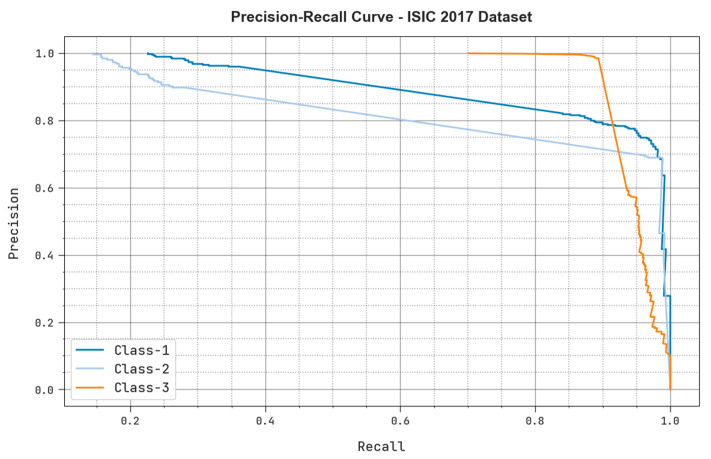
Precision recall analysis outcomes of the proposed MAFCNN-SCD system upon the ISIC 2017 dataset.

The precision-recall examination outcomes achieved by the proposed MAFCNN-SCD system under ISIC 2017 dataset are shown in Figure 8. The figure portrays that the proposed MAFCNN-SCD method produced high precision-recall values under each class label.

**Figure 9:**
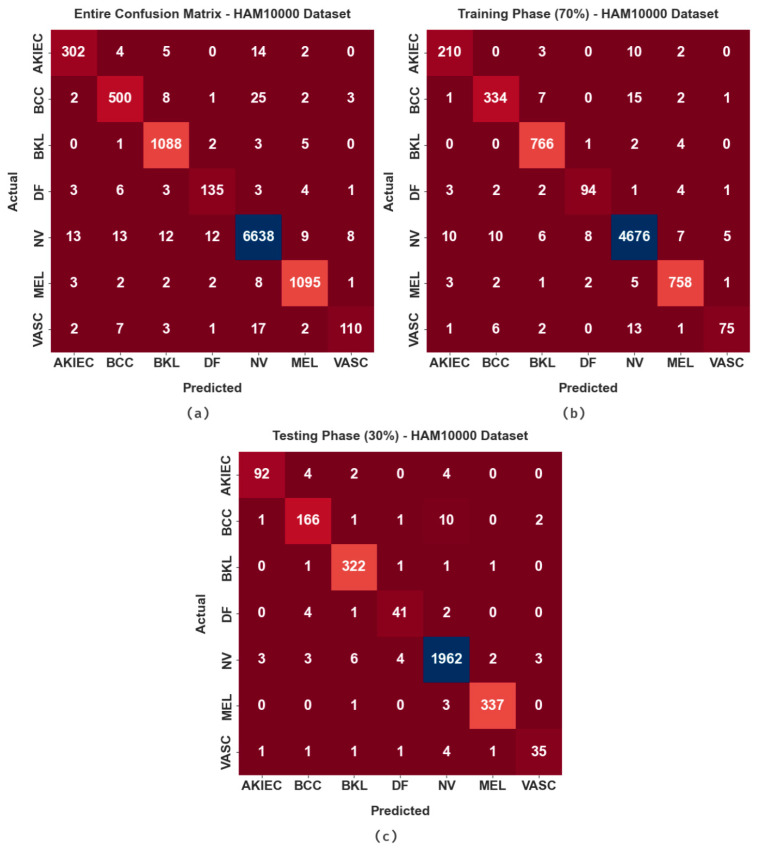
Confusion matrices of the proposed MAFCNN-SCD method under HAM10000 dataset (**a**) Entire database, (**b**) 70% of TR database, and (**c**) 30% of TS database.

The confusion matrices generated by the proposed MAFCNN-SCD algorithm on the HAM10000 dataset are exhibited in Figure 9. The figure shows that the MAFCNN-SCD approach proficiently recognized all seven types of skin cancer on the applied dermoscopic images.

Table 4 depicts the overall skin cancer classification results attained by the proposed MAFCNN-SCD method on the HAM10000 dataset. On the entire dataset, the proposed MAFCNN-SCD system achieved the average $accu_{y}$, $sens_{y}$, $spec_{y}$, $F_{score}$, and MCC values such as 99.39%, 92.25%, 99.49%, 93.08%, and 92.60%, correspondingly. At the same time, on 70% of the TR database, the MAFCNN-SCD algorithm attained the average $accu_{y}$, $sens_{y}$, $spec_{y}$, $F_{score}$, and MCC outcomes such as 99.42%, 92.40%, 99.51%, 93.27%, and 92.82%, correspondingly. Concurrently, on 30% of the TS database, the proposed MAFCNN-SCD algorithm achieved the average $accu_{y}$, $sens_{y}$, $spec_{y}$, $F_{score}$, and MCC values such as 99.34%, 91.92%, 99.44%, 92.67%, and 92.15%, correspondingly.

**Figure 10:**
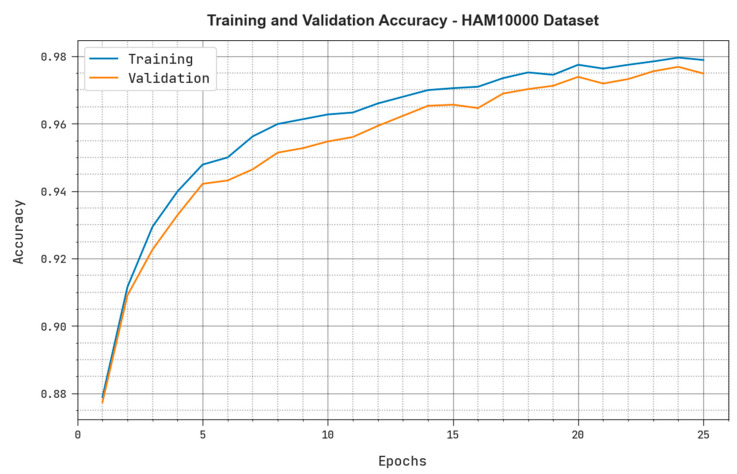
$TR_{acc}$ and $VL_{acc}$ outcomes of the proposed MAFCNN-SCD system upon the HAM10000 dataset.

Both $TR_{acc}$ and $VL_{acc}$ values acquired by the MAFCNN-SCD methodology under the HAM10000 dataset are exhibited in Figure 10. The simulation results confirm that the proposed MAFCNN-SCD approach obtained the maximum $TR_{acc}$ and $VL_{acc}$ values, whereas the $VL_{acc}$ values were better than the $TR_{acc}$ values.

**Figure 11:**
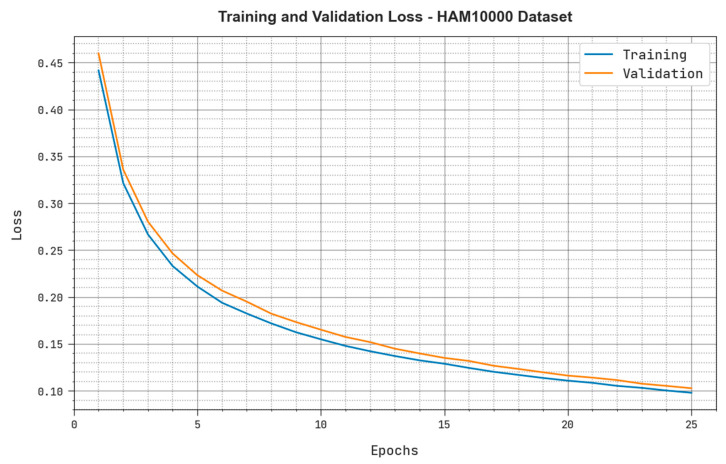
$TR_{loss}$ and $VL_{loss}$ results of the proposed MAFCNN-SCD system upon the HAM10000 dataset.

Both $TR_{loss}$ and $VL_{loss}$ values realized by the proposed MAFCNN-SCD system under the HAM10000 dataset are shown in Figure 11. The simulation results manifest that the presented MAFCNN-SCD approach obtained the least $TR_{loss}$ and $VL_{loss}$ values, while the $VL_{loss}$ values were lesser than the $TR_{loss}$ values.

**Figure 12:**
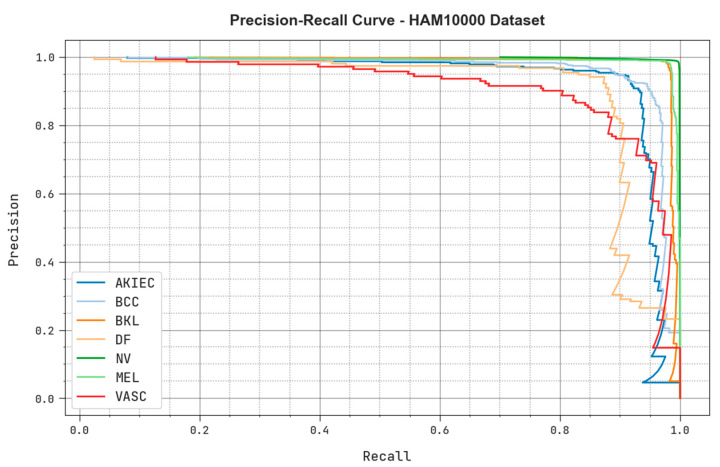
Precision recall analysis outcomes of the proposed MAFCNN-SCD system upon the HAM10000 dataset.

The precision-recall analysis results achieved by the MAFCNN-SCD system upon the HAM10000 dataset are exhibited in Figure 12. The figure demonstrates that the proposed MAFCNN-SCD method produced the maximum precision-recall values under each class label.

### 4.3. Discussion

Table 5 and Figure 12 report the comparative study outcomes of the MAFCNN-SCD model and other recent models on the ISIC 2017 dataset. The simulation results imply that the MobileNet method reached a poor performance while the MSVM model gained a slightly raised outcome. Next, the NB, KELM, and DenseNet169 models produced closer skin cancer classification performance. However, the proposed MAFCNN-SCD model accomplished the maximum performance with an accuracy of 92.22%.

Table 6 reports the comparative analysis outcomes achieved by the proposed MAFCNN-SCD method and other recent techniques on the HAM10000 dataset. The simulation outcomes imply that the NB methodology performed poorly, whereas the MobileNet approach acquired a slightly raised outcome.

Then, the KELM, MSVM, and DenseNet169 algorithms achieved closer skin cancer classification performance. But, the proposed MAFCNN-SCD model achieved the maximum performance. The comprehensive comparative analysis outcomes established the enhanced performance of the MAFCNN-SCD technique over other methodologies with maximum accuracy values such as 92.22% and 99.34% on the ISIC 2017 and HAM10000 datasets, respectively. These results establish the effectual skin cancer classification performance of the proposed MAFCNN-SCD method. The enhanced performance of the proposed model is due to the implementation of the HGSO algorithm in the hyperparameter tuning process.

## 5. Conclusions

In this study, a new MAFCNN-SCD approach has been modeled for skin cancer detection and classification from dermoscopic images. The major aim of the proposed MAFCNN-SCD technique is to identify and classify skin cancer from dermoscopic images. In the presented MAFCNN-SCD technique, the data pre-processing is performed initially. Next, the MAFNet methodology is applied as a feature extractor with the HGSO algorithm as a hyperparameter optimizer. Finally, the DBN method is exploited for skin cancer detection and classification. A sequence of experiments was conducted to showcase the supreme performance of the proposed MAFCNN-SCD approach. The comprehensive comparative analysis outcomes establish the enhanced performance of the MAFCNN-SCD technique over other methodologies with maximum accuracy values, such as 92.22% and 99.34% on the ISIC 2017 and HAM10000 datasets, respectively. Thus, the proposed model can be employed for the automated skin cancer classification process. In the future, the deep instance segmentation process can be incorporated to extend the detection rate of the MAFCNN-SCD technique to achieve low error rates in quantifying the skin lesion’s structure, boundary, and scale. To further increase the system’s performance, huge training datasets should be utilized to avoid under- and over-segmentation cases. Besides, the computation complexity of the proposed model should also be investigated in the future.

## Figures and Tables

**Table 1:** Details of the ISIC 2017 dataset [31].

ISIC 2017 Dataset		
Label	Class	No. of Samples
Class-1	Melanoma	374
Class-2	Seborrheic Keratosis	254
Class-3	Nevus	1372
Total Number of Dataset		2000

**Table 2:** Details on HAM10000 Dataset [32].

HAM10000 Dataset		
Description	Class	No. of Samples
Actinic Keratoses	AKIEC	327
Basal Cell Carcinoma	BCC	541
Benign Keratosis	BKL	1099
Dermatofibroma	DF	155
Melanocytic Nevus	NV	6705
Melanoma	MEL	1113
Vascular	VASC	142
Total No. of Dataset		10,082

**Table 3:** Skin cancer classification outcomes of the proposed MAFCNN-SCD system upon the ISIC 2017 dataset.

Labels	$Accu_{y}$	$Sens_{y}$	$Spec_{y}$	$F_{score}$	MCC
Entire Dataset					
Class-1	94.4	71.66	99.63	82.72	80.84
Class-2	95.75	68.9	99.66	80.46	79.56
Class-3	90.25	99.2	70.7	93.32	77.39
Average	93.47	79.92	90	85.5	79.26
Training Phase (70%)					
Class-1	94.71	72.9	99.74	83.77	82
Class-2	96.29	71.86	99.59	82.19	81.22
Class-3	91	99.18	72.49	93.86	78.77
Average	94	81.31	90.61	86.61	80.66
Testing Phase (30%)					
Class-1	93.67	68.75	99.39	80.21	78.1
Class-2	94.5	63.22	99.81	76.92	76.28
Class-3	88.5	99.25	66.83	92.02	74.32
Average	92.22	77.07	88.67	83.05	76.23

**Table 4:** Skin cancer classification outcomes of the proposed MAFCNN-SCD system upon the HAM10000 dataset.

Labels	$Accu_{y}$	$Sens_{y}$	$Spec_{y}$	$F_{score}$	MCC
Entire Dataset					
AKIEC	99.52	92.35	99.76	92.64	92.39
BCC	99.27	92.42	99.65	93.11	92.73
BKL	99.56	99	99.63	98.02	97.78
DF	99.62	87.1	99.82	87.66	87.47
NV	98.64	99	97.93	98.98	96.95
MEL	99.58	98.38	99.73	98.12	97.88
VASC	99.55	77.46	99.87	83.02	83.01
Average	99.39	92.25	99.49	93.08	92.6
Training Phase (70%)					
AKIEC	99.53	93.33	99.74	92.72	92.48
BCC	99.35	92.78	99.7	93.56	93.22
BKL	99.6	99.09	99.67	98.21	97.99
DF	99.66	87.85	99.84	88.68	88.51
NV	98.7	99.03	98.03	99.03	97.06
MEL	99.52	98.19	99.68	97.81	97.54
VASC	99.56	76.53	99.89	82.87	82.94
Average	99.42	92.4	99.51	93.27	92.82
Testing Phase (30%)					
AKIEC	99.5	90.2	99.83	92.46	92.24
BCC	99.07	91.71	99.54	92.22	91.73
BKL	99.47	98.77	99.56	97.58	97.29
DF	99.54	85.42	99.76	85.42	85.18
NV	98.51	98.94	97.7	98.87	96.7
MEL	99.74	98.83	99.85	98.83	98.68
VASC	99.54	79.55	99.83	83.33	83.2
Average	99.34	91.92	99.44	92.67	92.15

**Table 5:** Comparative analysis outcomes of the proposed MAFCNN-SCD model and other recent methodologies upon the ISIC 2017 dataset [33,34].

ISIC 2017 Dataset				
Methods	Accuracy	Sensitivity	Specificity	F-Score
MAFCNN-SCD	92.22	77.07	88.67	83.05
Naïve Bayes	89.77	74.7	84.02	81.37
KELM	88.04	77.03	84.49	83.2
MSVM	87.15	75.44	83.19	81.45
MobileNet	85.03	74.17	87.98	81.18
DenseNet169	89.42	76.83	86.28	83.27

**Table 6:** Comparative analysis outcomes of the proposed MAFCNN-SCD system and other recent algorithms upon the HAM10000 Dataset [33,34].

ISIC 2017 Dataset				
Methods	Accuracy	Sensitivity	Specificity	F-Score
MAFCNN-SCD	92.22	77.07	88.67	83.05
Naïve Bayes	89.77	74.7	84.02	81.37
KELM	88.04	77.03	84.49	83.2
MSVM	87.15	75.44	83.19	81.45
MobileNet	85.03	74.17	87.98	81.18
DenseNet169	89.42	76.83	86.28	83.27

## Data Availability

Data sharing is not applicable to this article as no datasets were generated during the current study.
